# Hierarchical investigating the predictive value of p53, COX2, EGFR, nm23 in the post-operative patients with colorectal carcinoma

**DOI:** 10.18632/oncotarget.13512

**Published:** 2016-11-23

**Authors:** Peng Du, Bin Xu, Dachuan Zhang, Yingjie Shao, Xiao Zheng, Xiaodong Li, Yuqi Xiong, Changping Wu, Jingting Jiang

**Affiliations:** ^1^ Department of Tumor Biological Treatment, The Third Affiliated Hospital, Soochow University, Changzhou 213003, Jiangsu, China; ^2^ Department of Oncology, The Third Affiliated Hospital, Soochow University, Changzhou 213003, Jiangsu, China; ^3^ Jiangsu Engineering Research Center for Tumor Immunotherapy, Changzhou 213003, Jiangsu, China; ^4^ The Second People's Hospital of Gansu Province, Lanzhou 730000, Gansu, China; ^5^ Department of Radiation Oncology, The Third Affiliated Hospital of Soochow University, Changzhou 213003, Jiangsu, China; ^6^ Institute of Cell Therapy, Soochow University, Changzhou, 213003, Jiangsu, China

**Keywords:** EGFR, nm23, post-operative, colorectal carcinoma

## Abstract

The aim of this study was to evaluate the correlations between p53, COX2, EGFR, nm23 expression and the progression free survival (PFS) of post-operative patients with colorectal carcinoma. Immunohistochemistry was used to detect the expression of p53, COX2, EGFR and nm23 in 459 specimens from colorectal carcinoma patients. Kaplan-Meier estimates, Cox proportional hazard regression analyses and hierarchical analyses were performed on the collected data. Kaplan-Meier estimates analysis suggested that EGFR expression was as a negative predictor, the median PFS of patients with EGFR high expression was 21.73 months, and the median PFS of patients with low EGFR expression was 57.83 months (*χ^2^*=20.880, *P*<0.001); nm23 expression was positive predictive factor for the prognosis of patients with colorectal carcinoma, the median PFS of patients with high nm23 expression was 37.77 months, and the median PFS was 21.47 months in the patients with low nm23 expression (*χ^2^*=7.364, *P*=0.007). Cox regression analysis revealed that comparing with the patients with low expression of EGFR, the patients with high EGFR expression were at higher risk of tumor progression (HR=1.667, *P*=0.004); Comparing with the patients with high nm23 expression, the patients with nm23 low expression had a higher risk of tumor progression (HR=0.412, *P*<0.001); and the risk of tumor progression was higher in the patients with high EGFR expression and low nm23 expression (HR=0.245, *P*<0.001). Hierarchical analysis showed that EGFR expression mainly correlates with the PFS of TNM stage I-II colorectal cancer patients, the median PFS was 33.53 months in the TNM stage I-II colorectal cancer patients with high EGFR expression patients; The median PFS of the TNM stage I-II colorectal cancer patients with low EGFR expression was 70.43 months (*χ^2^*=9.530, *P*=0.002); The median PFS was 19.2 months in the TNM stage III-IV colorectal cancer patients with high expression EGFR, the PFS of the TNM stage III-IV colorectal cancer patients with low EGFR expression was 37.87 months (*χ^2^*=7.97, *P*=0.005). nm23 expression mainly correlates with the PFS of TNM stage III-IV colorecatal cancer patients. The median PFS was 47.27 months in TNM stage I-II colorectal cancer patients with nm23 high expression, the median PFS was 48.85 months in TNM stage I-II colorectal cancer patients with low nm23 expression (*χ^2^*=0.101, *P*=0.750); The median PFS was 28.8 months in TNM stage III-IV colorectal cancer patients with nm23 high expression, the median PFS was 14.7 months in TNM stage III-IV colorectal cancer patients with low nm23 expression (*χ^2^*=13.213, *P*<0.001). EGFR is mainly a predictive factor for the prognosis of post-operative patients with TNM stage I-II colorectal cancer, and nm23 is important for predicting the prognosis of patients with stage III-IV, and EGFR and nm23 could be as predictor of combination.

## INTRODUCTION

Colorectal carcinoma (CRC) is one of the most common malignant tumors worldwide [1–3], and it is also the third most common malignant disease in Asia [4]. Previous studies have shown that various biomarkers, such as p53, COX2, EGFR and nm23, were involved in multiple stages of tumor development [4–15]. These biomarkers, most of which are oncogenes and tumor suppressors, play a crucial role in carcinoma formation [3, 15, 16]. Therefore, they were used for monitoring the progression of tumors and guiding targeting therapies. So far, the prognosis of patients with CRC depends mainly on TNM staging system. However, the patients with similar disease features can manifest various survival results, Therefore, it is important to find molecular prognostic factors that can be useful to identify patients who are at high risk of progression and to individualize treatment [17]. Moreover, one biomarker often fails to predict the prognosis of cancer patients [18]. There is still a lack of better biomarker combinations to predict the prognosis in different stages of carcinoma. Thus, we enrolled 459 patients with colorectal cancer in this study and investigated hierarchically the predictive value of clinicopathological biomarkers for the prognosis of post-operative patients with colorectal carcinoma.

## RESULTS

### The expression of P53, COX2, EGFR and nm23 in colorectal cancer tissues

**Figure 1:**
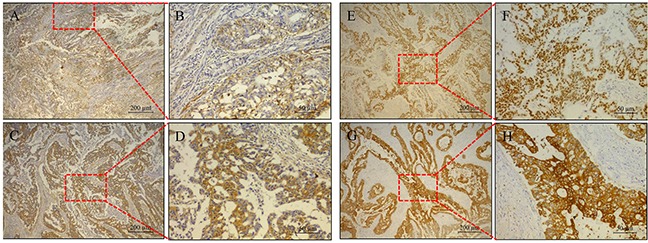
Immunohistochemistry results The expression of EGFR **A.** and **B.**, nm23 **C.** and **D.**, p53 **E.** and **F.**, and COX2 **G.** and **H.** in patients derived sections.

Immunohistochemistry results showed that P53, COX2, EGFR and nm23 were proportionally expressed in colorectal cancer tissues, and the positive expression of p53, COX2, EGFR and nm23 were shown in Figure 1. In the patients with colorectal carcinoma, EGFR expression accounted for 53.59%, COX2 expression accounted for 84.1%, p53 expression accounted for 58.52%, nm23 expression accounted for 67.25%. Correlations between the expression of them in colorectal cancer and age, gender, Body Mass Index (BMI), pathological grade, tumor size, lymphatic metastasis, TNM stage were analyzed. The results showed that the correlation between EGFR expression and TNM stage (*χ^2^*=9.049, *P*=0.003) as well as gender (*χ^2^*=4.956, *P*=0.026) were statistically significant, but not others (Table 1).

**Table 1:** Relationships between clinicopathological characteristics and the expression of p53, COX2, EGFR and nm23

Clinical parameter		EGFR expression level				COX2 expression level				p53 expression level				nm23 expression level			
		Low	High	χ^2^	*P*	Low	High	χ^2^	*P*	Low	High	χ^2^	*P*	Low	High	χ^2^	*P*
Age(Years)	<60	121	139	0.004	0.948	41	219	0.008	0.928	101	158	1.521	0.218	93	167	2.487	0.115
	≥60	92	107			32	167			89	110			57	141		
Gender	female	76	113	4.956	**0.026**	24	165	2.469	0.116	76	112	0.147	0.701	62	127	0.000	0.984
	male	137	133			49	221			114	156			88	181		
BMI	<25	129	108	0.003	0.958	22	215	0.475	0.491	98	139	0.103	0.748	88	148	0.267	0.605
	≥25	46	38			10	74			36	47			34	50		
pathology grading	I-II	121	125	1.649	0.199	42	204	0.542	0.462	113	133	4.336	0.037	81	165	0.007	0.931
	III-IV	92	121			31	182			77	135			69	143		
Tumor size(cm)	<8cm	109	124	0.014	0.905	42	191	1.542	0.214	96	136	0.000	0.992	73	160	0.351	0.554
	≥8cm	104	121			31	194			93	132			76	148		
Lymphatic metastasis	No	101	103	1.162	0.281	35	169	0.707	0.400	87	116	0.262	0.608	67	136	0.038	0.845
	Yes	112	140			36	216			102	150			81	171		
TNM stage	I-II	104	86	9.049	**0.003**	35	155	1.536	0.215	85	105	1.415	0.234	59	130	0.344	0.558
	III-IV	109	160			38	231			105	163			91	178		

* lack of partial data

### Relationships between p53, COX2, EGFR, nm23 expression and PFS of patients

**Figure 2:**
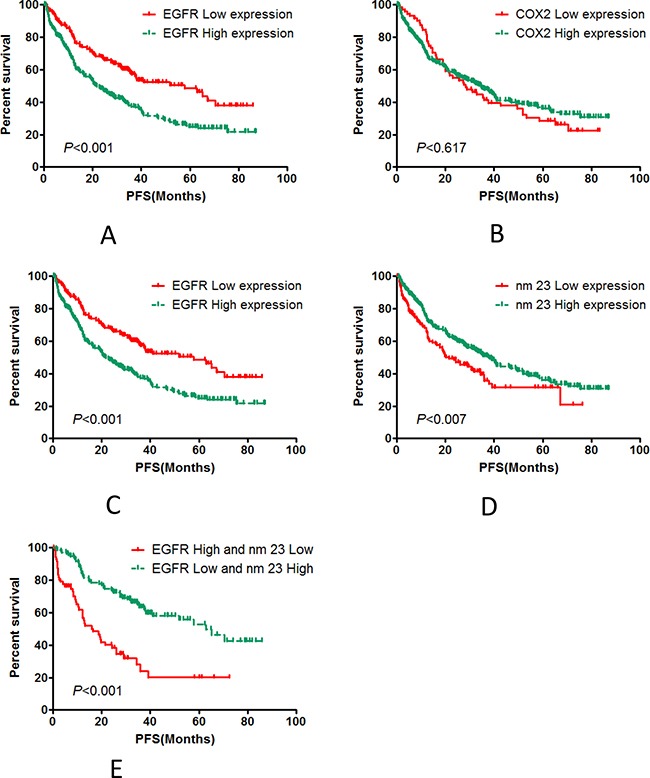
Statistical analyses for the correlation between PFS and the protein expression of p53, COX2, EGFR, nm23, and EGFR&nm23 combined The expression of p53 **A.** and COX2 COX2 **B.** were not related to PFS. The expression of EGFR **C.** and nm23 **D.** were highly associated with PFS. And the PFS of patients with low EGFR and high nm23 expression **E.** was significantly different than those with high EGFR and low nm23 expression.

We performed statistical analyses on the correlations between progression free survival (PFS) and the patients' gender, age, BMI, pathological grade, tumor size, lymphatic metastasis, TNM stage, expression of EGFR, COX2, p53, nm23, and EGFR&nm23 combined expression. pathological grade, TNM stage, EGFR and nm23 expression had statistical significance to the PFS of post-operative patients with colorectal carcinoma (*P*<0.05). The median PFS of pathological grade I-II colorectal cancer patients was 39.83 months, the median PFS of pathological grade III-IV colorectal cancer patients was 22.87 months (*χ^2^*=7.270, *P***=**0.007). The median PFS was 40.40 months in the TNM I-II colorectal cancer patients, the PFS of the TNM stage III-IV colorectal cancer patients was 23.53 months (*χ^2^*=11.382, *P*=0.001), EGFR expression was as a negative predictor, the median PFS of patients with EGFR high expression was 21.73 months, and the median PFS of patients with low EGFR expression was 57.83 months (*χ^2^*=20.880, *P*<0.001); nm23 expression was positive predictive factor for the prognosis of patients with colorectal carcinoma, the median PFS of patients with high nm23 expression was 37.77 months, and the median PFS was 21.47 months in the patients with low nm23 expression (*χ^2^*=7.364, *P*=0.007). Interestingly, the median PFS was 62.93 months in the patients with low EGFR and high nm23 expression, the median PFS was 16.38 months in the patients with high EGFR and low nm23 expression(*χ^2^*=30.396, *P*<0.001). but other clinicopathological parameters had not statistical significance (Table 2 and Figure 2).

**Table 2:** Correlations between the progression free survival (PFS) and Clinicopathological parameters

Clinical parameters		Cases	Median PFS (Months)	Log-Rank χ^2^	P value
Gender	Male	270	34.40	0.073	0.787
	Female	189	31.87		
Age(years)	≤60	260	33.53	0.098	0.754
	>60	199	32.00		
BMI	≤25	237	58.63	0.034	0.854
	>25	84	49.03		
Pathology grade	I-II	246	39.83	7.270	**0.007**
	III-IV	213	22.87		
Tumor size	≤8cm	233	30.67	1.594	0.207
	>8cm	225	37.37		
Lymph node	Negative	204	34.70	0.477	0.490
	Positive	252	28.37		
TNM stage	I-II	190	40.40	11.382	**0.001**
	III-IV	269	23.53		
EGFR expression level	Low	213	57.83	20.880	**<0.001**
	High	246	21.73		
COX2 expression level	Low	73	28.37	0.250	0.617
	High	386	34.57		
p53 expression level	Low	190	35.23	1.604	0.205
	High	268	30.53		
nm23 expression level	Low	150	21.47	7.364	**0.007**
	High	308	37.77		
EGFR & nm23 combined expression level	EGFR high & nm23 low	73	16.38	30.396	**<0.001**
	EGFR low & nm23 high	136	62.93		

* lack of partial data

### Cox regression results

Cox regression analysis revealed that comparing with the patients with pathological grade I-II, patients with pathological grade III-IV had higher tumor progression risk (*HR*=1.521, 95% *CI*:1.068-2.167, *P*=0.02); patients with TNM stage I-II colorectal carcinoma *vs.* patients with TNM stage III-IV colorectal carcinoma, the latter is at higher risk of tumor progression (*HR*=1.604, 95% *CI*: 1.108-2.321, *P*=0.012), and TNM staging is an independent prognostic factor; Comparing with the patients with low expression of EGFR, the patients with high EGFR expression were at higher risk of tumor progression (*HR*=1.667, 95% *CI*: 1.177-2.362, *P*=0.004); Comparing with the patients with high nm23 expression, the patients with nm23 low expression had a higher risk of tumor progression (*HR*=0.412, 95% *CI*: 0.288-0.591, *P*<0.001); the risk of tumor progression was higher in the patients with high EGFR expression and low nm23 expression (*HR*=0.245, 95% *CI*: 0.142-0.426, *P*<0.001), (Table 3 and Table 4).

**Table 3:** Cox regression model analysis for Clinicopathological parameters

Clinicopathological paramerers	Multivariate		
	HR	95% CI	P value
Age(Years)			
>=60/<60	1.020	0.722-1.439	0.912
Gender			
Male/Female	0.860	0.612-1.209	0.386
Body Mass Index			
>=25/<25	1.041	0.706-1.533	0.841
Pathology grade			
III-IV/I-II	1.521	1.068-2.167	**0.020**
Tumor size			
>=8cm/<8cm	0.802	0.571-1.127	0.203
TNM stage			
III-IV/I-II	1.604	1.108-2.321	**0.012**
EGFR expression level			
High/Low	1.667	1.177-2.362	**0.004**
COX2 expression level			
High/Low	1.794	0.924-3.481	0.084
p53 expression level			
High/Low	1.186	0.829-1.697	0.350
nm23 expression level			
High/Low	0.412	0.288-0.591	**<0.001**

**Table 4:** Cox regression model analysis for Clinical parameters and combined pathology index

Clinicopathological paramerers	Multivariate		
	HR	95% CI	P value
Age(Years)			
>=60/<60	0.858	0.509-1.446	0.565
Gender			
Male/Female	0.806	0.468-1.389	0.438
Body Mass Index			
>=25/<25	1.027	0.575-1.833	0.929
Pathology grade			
III-IV/I-II	1.903	1.114-3.252	**0.018**
Tumor size			
>=8cm/<8cm	1.032	0.604-1.762	0.909
TNM stage			
III-IV/I-II	1.651	0.924-2.952	0.091
COX2 expression level			
High/Low	1.219	0.469-3.173	0.685
p53 expression level			
High/Low	1.319	0.789-2.207	0.291
Combined with EGFR & nm23			
EGFR low & nm23 high/EGFR high & nm23 low	0.245	0.142-0.426	**<0.001**

### Hierarchical analyses about EGFR and nm23 expression in the TNM stage I-II and III-IV colorectal cancer patients

**Figure 3:**
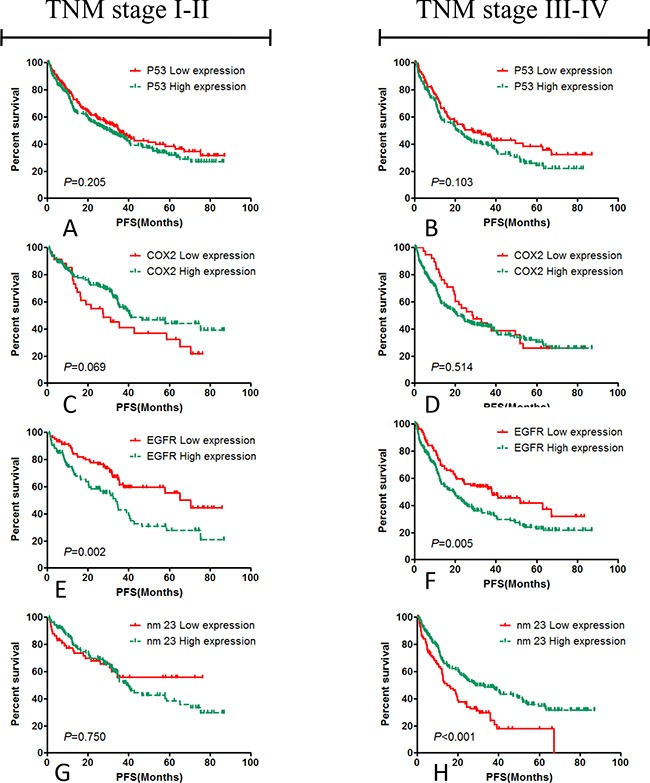
Hierarchical analysis of the correlation between PFS and the expression of p53, COX2, EGFR, nm23 in the patients with TNM stage I-II and III-IV p53 **A.** and **B.** and COX2 **C.** and **D.** had not statistical significance to the PFS of post-operative patients with colorectal carcinoma, instead of EGFR **E.** and **F.** and nm23 **G.** and **H.**, and the results suggested that high EGFR expression promoted the cancer progression of the post-operative patients with colorectal cancer in TNM stage I-II, and the nm23 expression mainly influenced the PFS of the patients with TNM stage III-IV.

Hierarchical analysis showed that EGFR expression mainly affects the PFS of TNM stage I-II colorectal cancer patients, the median PFS was 33.53 months in the TNM stage I-II colorectal cancer patients with high EGFR expression patients, The median PFS was 70.43 months in the TNM stage I-II colorectal cancer patients with low EGFR expression (*χ^2^*=9.530, *P*=0.002); The median PFS was 19.2 months in the TNM stage III-IV colorectal cancer patients with high expression EGFR, the PFS of the TNM stage III-IV colorectal cancer patients with low EGFR expression was 37.87 months (*χ^2^*=7.97, *P*=0.005). nm23 expression mainly affects the PFS of TNM stage III-IV colorecatal cancer patients. The median PFS was 47.27 months in TNM stage I-II colorectal cancer patients with nm23 high expression, the median PFS was 48.85 months in TNM stage I-II colorectal cancer patients with low nm23 expression (*χ^2^*=0.101, *P*=0.750); The median PFS was 28.8 months in TNM stage III-IV colorectal cancer patients with nm23 high expression, the median PFS was 14.7 months in TNM stage III-IV colorectal cancer patients with low nm23 expression (*χ^2^*=13.213, *P*<0.001). These results were shown in Table 5 and Figure 3.

**Table 5:** Hierarchical analysis of the relationships between PFS and the expression of p53, COX2, EGFR, nm23 in the patients with TNM stage I-II and III-IV

Hierarchical define	Clinical parameters		Cases	Median/Mean PFS (Months)	Log-Rank χ^2^	P value
TNM stage I-II						
	EGFR expression level	Low	104	70.43	9.530	**0.002**
		High	86	33.53		
	COX2 expression level	Low	35	27.53	3.311	0.069
		High	155	40.87		
	p53 expression level	Low	85	39.83	0.132	0.717
		High	105	40.40		
	nm23 expression level	Low	59	48.85^#^	0.101	0.750
		High	130	47.27^#^		
TNM stage III-IV						
	EGFR expression level	Low	109	37.87	7.97	**0.005**
		High	160	19.2		
	COX2 expression level	Low	38	28.37	0.426	0.514
		High	231	21.03		
	p53 expression level	Low	105	28.37	2.655	0.103
		High	163	21.03		
	nm23 expression level	Low	91	14.7	13.213	**<0.001**
		High	178	28.8		

### Hierarchical analyses about EGFR and nm23 expression in the pathological grade I-II and III-IV colorectal cancer patients

**Figure 4:**
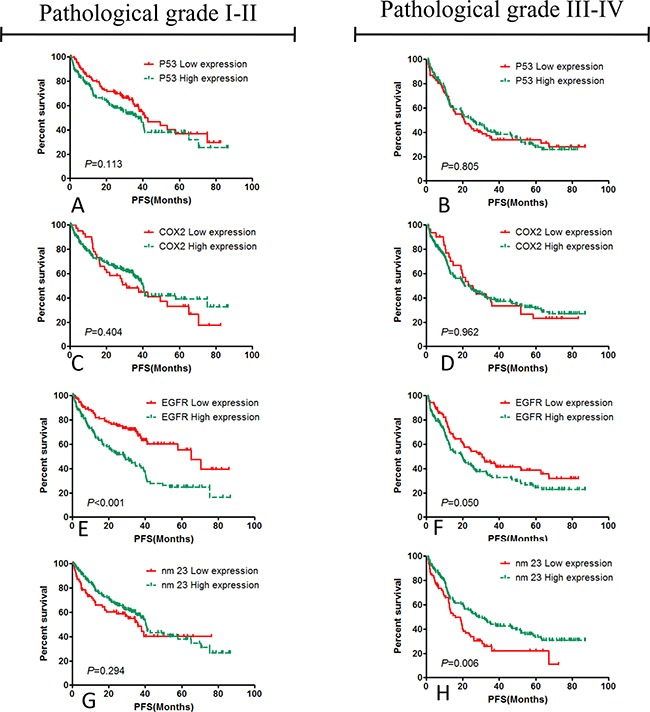
Hierarchical analysis of the relationships between PFS and the expression of EGFR, COX2, p53, nm23 in the patients with pathological grade I-II and III-IV p53 **A.** and **B.** and COX2 **C.** and **D.** had not statistical significance to the PFS of post-operative patients with colorectal carcinoma, instead of EGFR **E.** and **F.** and nm23 **G.** and **H.**, and the results suggested that high EGFR expression promoted the cancer progression of the post-operative patients with colorectal cancer in pathological grade I-II, and the nm23 expression mainly influenced the PFS of the patients with pathological grade III-IV.

Hierarchical analysis showed that EGFR expression mainly affects the PFS of pathological grade I-II colorectal cancer patients, the median PFS of pathological grade I-II colorectal cancer patients with EGFR high expression was 28.37 months, the median PFS of pathological grade I-II colorectal cancer patients with low EGFR expression was 65.07 months (*χ^2^*=18.766, *P***<**0.001). The median PFS was 19.6 months in pathological grade III-IV colorectal cancer patients with high EGFR expression, the median PFS in pathological grade III-IV colorectal cancer patients with low EGFR expression was 30.67 months (*χ^2^*=3.846, *P*=0.05). nm23 Expression mainly affects PFS of the pathological grade III-IV colorectal cancer patients, the median PFS was 40.40 months in pathological grade I-II colorectal cancer patients with high nm23 expression, and the median PFS was 35.93 months in pathological grade I-II colorectal cancer patients with low nm23 expression (*χ^2^*=1.102, *P***=**0.294). the median PFS was 28.53 months in pathological grade III-IV colorectal cancer patients with high nm23 expression, the median PFS was 15.93 months in pathological grade III-IV colorectal cancer patients with low expression of nm23 (*χ^2^*=7.699, *P*=0.006). These results were shown in Table 6 and Figure 4.

**Table 6:** Hierarchical analysis of the relationships between PFS and the expression of p53, COX2, EGFR, nm23 in the patients with pathological grade I-II and III-IV

Hierarchical define	Clinical parameters		Cases	Median/Mean PFS (Months)	Log-Rank χ^2^	P value
Pathology grade I-II						
	EGFR expression level	Low	121	65.07	18.766	**<0.001**
		High	125	28.37		
	COX2 expression level	Low	42	31.00	0.697	0.404
		High	204	40.07		
	p53 expression level	Low	113	40.87	2.511	0.113
		High	133	37.37		
	nm23 expression level	Low	81	35.93	1.102	0.294
		High	165	40.40		
Pathology grade III-IV						
	EGFR expression level	Low	92	30.67	3.846	**0.050**
		High	121	19.6		
	COX2 expression level	Low	31	25.53	0.002	0.961
		High	182	21.03		
	p53 expression level	Low	77	20.77	0.061	0.805
		High	135	25.53		
	nm23 expression level	Low	69	15.93	7.699	**0.006**
		High	143	28.53		

## DISCUSSION

Several studies have provided evidence that some of biomarkers may be useful in identifing the risk for post-operative patients with CRC and in defining patients who may get benefit from adjuvant chemotherapy [17, 19]. Some of them, such as p53 and COX2, play a critical role in carcinogenesis [20, 21]. It has been confirmed that p53 and COX2 mutations could induce carcinogenesis [4, 14], and COX2 could increase the risk of adenoma recurrence [15]. The mechanisms included inhibiting the apoptosis of cancer cells, promoting tumor angiogenesis, inhibiting the immunity of the organism, and increasing the invasion and metastasis of tumors^[22–25]^. However, in this study, we did not find correlations between p53, COX2 expression and the prognosis of colorectal cancer, which is inconsistent with the results from previous studies [8, 9, 26]. The reason for this could be that p53, COX2 only promotes carcinogenesis, instead of the progression of the disease.

In this study, we investigated the correlations between the progression free survival and patients' gender, age, body mass index, pathology stage, tumor size, lymphatic metastasis, TNM stage, EGFR, COX2, p53, nm23, Combined with EGFR and nm23, and evaluated mainly the possible prognostic and predictive values of p53, COX2, EGFR and nm23. We found that a statistically significant correlation between PFS and Pathology grade, TNM stage, EGFR and nm23 expression. To date, pathologic grading and TNM staging are still used as predictors for prognosis of tumor patients, and plays a role in patient management, including informing prognosis and therapeutic decision making. However, several studied suggest that there may also be significant variation in prognosis of patients within even so the same tumor stage [27, 28]. Therefore, some biomarkers are getting more and more attention, and so far biomarkers based on cancer biology have already risen to important position in the research of malignant tumors and clinical management [18]. We found that EGFR and nm23 expression were closely related to the prognosis of patients: the risks of recurrence and metastasis were higher for the patients with high EGFR expression and early TNM stage I-II; the PFS was worse for the patients with low nm23 expression and TNM stage III-IV; the risk of progression will further increase in the patients with high EGFR expression and low nm23 expression. Previous studies already suggested that EGFR played an important role in angiogenesis of early tumor, which is associated with the progression of colorectal carcinoma, and is a widely used prognostic factor [6, 29–31]. The over expression of EGFR promotes the metastasis, invasion. The mechanisms mainly included promoting tumor angiogenesis and proliferation of tumor cells that plays a critical role in the early carcinogenesis and progression. EGFR downstream signal transduction pathways are primarily two: one is the Ras/Raf/MEK/ERK- MAPK pathway, and the other is the PI3K/AKT/mTOR pathway [32–38]. Some study has also demonstrated that anti-EGFR antibody could improve the prognosis of colorectal carcinoma [39]. The nm23 gene and the related proteins have NDPK activities, which can change the energy metabolism of the cells, and then influence the development and occurrence of the tumor [10–12, 40]. It has been confirmed that the down-regulation of nm23 gene was an early event in tumor progression, in which the structure and function of the gene was changed and it had direct impact on the cell configuration, mobility, adhesion and spindle formation in mitosis. nm23 gene could inhibit the metastasis of tumor cells, so that nm23 gene expression was negatively correlated with lymph node metastasis, and was positively correlated with PFS [10–12, 40], and our results also indicted that nm23 plays mainly an important role in TNM stage III-IV. So the detection of nm23 gene expression level can be used as an important indicator of whether the metastasis of the tumor.

So far, there is no study with large sample size and multi-center to verify the specific roles of these biomarkers in different TNM stages, as well as evaluated which marker was better for prognosis, which combination of predictive markers was more predictive, in order to guide the clinical workers to select the suitable time and patients for targeted therapies. This study suggested that EGFR was an important predictive factor for the prognosis of the post-operative patients with colorectal carcinoma TNM stage I-II, and nm23 is important for predicting the prognosis of the patients with stage III-IV; it is better that EGFR and nm23 are as predictor of combination. These results would be helpful for evaluation of prognosis and choosing suitable therapies for the post-operative patients with colorectal carcinoma.

## PATIENTS AND METHODS

### Patients

A total of 1086 patients with CRC who underwent surgery at The Third Affiliated Hospital of Soochow University were enrolled in this study from January 2003 to October 2010. All surgical specimens with the possibility of a diagnosis of CRC were additionally reviewed by two pathologists. The study was approved by the Research Ethics Committee of the Third Affiliated Hospital of Soochow University. However, some patients who did not meet the inclusion criteria, or had other tumors were excluded from the study. Some cases were excluded in the procedure of follow-up. Finally, all 459 patients, including 270 males and 189 females, aged 29-83 years with a median age of 55 years, were enrolled. Exclusion criteria include: 1. we cannot contact with patients or their families to know about the patient's conditions; 2. patients suffered from other tumors or chronic diseases, and they died from these diseases or other accidents. The inclusion criteria: patients didn't have other chronic diseases and distant metastasis; they had complete pathological data and medical history; they didn't receive radiotherapy, chemotherapy or other treatment methods before the surgery.

### Immunohistochemical staining

Immunohistochemical staining was performed using streptavidin-perosidase (SP) method. Slides were created by transverse sectioning (4μm). The paraffin-embedded slides were deparaffinated with xylene and rehydrated through a series of ethanol solutions with decreasing concentrations. Endogenous peroxidase activity was blocked by 0.3% H_2_O_2_ in methanol at room temperature for half an hour. Thereafter antigen retrieval was achieved by heating the slides in a phosphate-buffered saline (pH 6.0) for 20 minutes and cooling them in the same buffer for 10 minutes. Then the slides were incubated with a diluted primary antibody (mouse anti-COX2, clone COX229, 1:100 dilution, Zymed Laboratories Inc, South San Francisco, Calif; mouse antihuman p53, DO7, Dako, Denmark, 1:50 dilution; rabbit anti-EGFR, MaiXin Biotechnologies, China, 1:400 dilution; anti-human nm23, Dako, Denmark,1:50 dilution.) overnight at 4°C. Then an undiluted secondary antibody was applied for 30 minutes. Rinsing between steps was performed with phosphate buffered saline. The slides were developed with diaminobenzidine and counterstained with Bayers' haematoxylin. After that the slides were dehydrated and mounted with cover slips.

### Immunohistochemistry (IHC) scores

The results were scored by evaluating the percentage of tumor cells that had nuclear staining for p53, COX2, EGFR and nm23. Protein expression was classified as negative if <10% of tumor cells were stained [17, 41]. Otherwise they were categorized as positive. Positive expression was assorted further according to the expression levels: mild expression (from ≥ 10% to < 25% positive tumor cells), moderate expression (from ≥ 25% to < 50% positive tumor cells), and high expression (≥ 50% positive tumor cells), with scores ranging from 0 to 6. A score of 0 meant no staining, a score of 1 or 2 indicated weak staining, a score of 3 or 4 meant moderate staining, and a score of 5 or 6 suggested strong staining. However, during statistical analysis, expression was assorted synoptically as either negative or positive.

### Statistical analyses

The relationships between the PFS of post-operative patients and the expression of p53, COX2, EGFR and nm23 and other clinical parameters were analyzed by SPSS 19.0 statistical software. Kaplan-Meier survival estimates and Cox regression analysis were used for calculating PFS (dependent variable). And different prognostic factors, including the tested markers, were used to depict their independent effects on the survival. Contingency tables and chi-squared test (Pearson) were used to evaluate the relationships between the expression of p53, COX2, EGFR, nm23 and other factors, such as tumor stage, grade of tumor differentiation, gender, age, etc. All data were described by the hierarchical analysis. Differences were considered as significant when *P* < 0.05.
